# Neuromotor Speech Recovery Across Different Behavioral Speech Modifications in Individuals Following Facial Transplantation

**DOI:** 10.3389/fneur.2020.593153

**Published:** 2021-01-06

**Authors:** Marziye Eshghi, Bridget J. Perry, Brian Richburg, Hayden M. Ventresca, Bohdan Pomahac, Jordan R. Green

**Affiliations:** ^1^Speech and Feeding Disorders Lab, MGH Institute of Health Profession, Boston, MA, United States; ^2^Plastic Surgery, Brigham and Women's Hospital, Harvard Medical School, Boston, MA, United States

**Keywords:** facial transplantaion, speech modifications, kinematics, neural recovery, speed of movement, range of movement

## Abstract

Despite signs of facial nerve recovery within a few months following face transplantation, speech deficits persist for years. Behavioral speech modifications (e.g., slower-than-normal speaking rate and increased loudness) have shown promising potential to enhance speech intelligibility in populations with dysarthric speech. However, such evidence-based practice approach is lacking in clinical management of speech in individuals with facial transplantation. Because facial transplantation involves complex craniofacial reconstruction and facial nerve coaptation, it is unknown to what extent individuals with face transplant are capable of adapting their motor system to task-specific articulatory demands. The purpose of this study was to identify the underlying articulatory mechanisms employed by individuals with face transplantation in response to speech modification cues at early and late stages of neuromotor recovery. In addition, we aimed to identify speech modifications that conferred improved speech clarity. Participants were seven individuals who underwent full or partial facial vascularized composite allografts that included lips and muscles of facial animation and were in early (~2 months) or late (~42 months) stages of recovery. Participants produced repetitions of the sentence “Buy Bobby a puppy” in normal, fast, loud, and slow speech modifications. Articulatory movement traces were recorded using a 3D optical motion capture system. Kinematic measures of average speed (mm/s) and range of movement (mm^3^) were extracted from the lower lip (± jaw) marker. Two speech language pathologists rated speech clarity for each speaker using a visual analog scale (VAS) approach. Results demonstrated that facial motor capacity increased from early to late stages of recovery. While individuals in the early group exhibited restricted capabilities to adjust their motor system based on the articulatory demands of each speech modification, individuals in the late group demonstrated faster speed and larger-than-normal range of movement for loud speech, and slower speed and larger-than-normal range of movement for slow speech. In addition, subjects in both groups showed overreliance on jaw rather than lip articulatory function across all speech modifications, perhaps as a compensatory strategy to optimize articulatory stability and maximize speech function. Finally, improved speech clarity was associated with loud speech in both stages of recovery.

## Introduction

With over 40 facial transplantation surgeries completed worldwide, this procedure is now considered an effective reconstructive option for restoring a patient's facial appearance and oromotor functions after a traumatic injury. Despite evidence of facial nerve recovery within a few months post-surgery (1–3), speech deficits persist for years (4, 5). Although the existing literature on long-term outcomes is sparse, improvement of neuromotor function following facial transplantation has been documented in at least three studies (2, 6, 7). Recently, Tasigiorgos et al. (6) conducted a 5-year follow-up of motor recovery in six patients with full and partial facial transplants using Daniels and Worthingham Muscle Testing. Findings of their study revealed that motor function showed rapid improvement during the 1st year after transplantation and continued to improve at a slower rate after the 1st year. At 5 years of follow-up, motor recovery had reached a mean of 60% of maximal motor function. Similarly, De Letter and colleges (2) reported that facial motor function improved over 38 months post-surgery as indicated by gains in lip motor function scores and increased muscle activation levels based on electromyography (EMG) during a speech task (i.e., a sentence completion task). In addition to these longitudinal studies, a case study conducted by Grigos et al. (7) reported increases in vertical jaw and lip movements during speech and non-speech (e.g., lip opening, closure, retraction, and protrusion) tasks over 13 months post-transplant. The same study also reported several negative findings such that the same gains were not seen for lip spreading, and jaw and lip movement variability were greater than the controls, which may in part explain continued mild functional speech impairments in this population.

One untested hypothesis is that the impact of these residual facial motor impairments on speech may be minimized using speech modification techniques that are commonly used in speech treatment, such as decreasing rate, increasing loudness, or intentionally speaking as clearly as possible. These modifications have been demonstrated to enhance speech intelligibility in a wide-variety of speech impairments (8–17). Each speech modification differs in the demands they place on the speech motor system. As compared to the normal (habitual) speech, increasing the rate of speech (i.e., fast speech) tends to elicit greater movement speeds but smaller articulatory displacements (18–23). During fast speech, speakers typically truncate articulatory displacement rather than alter the speed of movement as a strategy to economize effort (21, 24, 25). Slow speech, in contrast, tends to elicit larger articulatory displacement, slower movement speed, and longer movement duration (26, 27). Some research suggests that the longer duration associated with slow speech enhances articulatory precision (28, 29) or phoneme distinctiveness (9, 30–32) and improves speech intelligibility (33, 34); however, findings are mixed. Several studies have found that slow speech did not promote more precise articulation, but rather clear speech allowed speakers to maintain control over jaw opening movements and improved speech intelligibility (10, 35).

Much of the research on loud speech interventions has been focused on testing the efficacy of the Lee Silverman Voice Treatment (LSVT^R^) program (36, 37), which was initially developed to improve speech in individuals with Parkinson disease (PD). Loud speech, in comparison to normal speech, elicits global gains across the speech system such as larger articulator displacements and faster movement speeds (9, 17, 38–41), greater respiratory drive (42, 43), greater subglottal air pressure (44), and improved vocal fold function (45). These physiologic changes can have the overall effect of enhancing speech accuracy, speech clarity, and speech intelligibility (12, 44, 46–48).

To our knowledge, the efficacy of speech modification techniques for improving speech following facial transplantation has not been evaluated. Although research exploring the impact of facial nerve repair on speech motor control is sparse (7), impairments in motor control for facial expression have been reported for populations undergoing unilateral facial nerve coaptation (49). A recent systematic review and meta-analysis exploring recovery of facial movement following masseteric facial nerve transfer in patients with facial paralysis found that the mean time to initial movement of smile excursion was 4.95 months (49). Additionally, differences in recovery of purposeful vs. spontaneous facial expressions have been documented, with spontaneous smiles found to be present in only 25/108 (23%) patients (49). Therefore, as a result of facial nerve coaptation during surgery, individuals after face transplant may exhibit limited ability to perform different motoric demands of fast, loud, and slow speech modifications, particularly in the early stages of recovery. As such, examining the efficacy of behavioral speech modifications in deriving articulatory functional gain in individuals with facial transplants at different time points in recovery to improve speech clarity and intelligibility is warranted.

The purpose of this study was to determine (1) the extent to which patients recovering from facial transplantation surgery can adapt their motor system to various articulatory demands of different speech modifications using measures of facial biomechanics, and (2) the comparative effects of these adaptations on speech clarity. Based on limited available literature, we hypothesized that motor adaptation to articulatory demands of speech modifications will be restricted during the early stages of neural recovery and that loud speech would confer improved speech clarity. This information is needed to identify optimal articulatory strategies to promote oromotor functional gain throughout the course of neural recovery and to provide an assessment technique to monitor the rate of neural recovery following facial transplant surgery.

## Method

### Participants

Seven participants who had undergone full or partial facial vascularized composite allografts were included in this study. Participants were at varying phases of recovery. Three (three males) participants were between 0 and 3 months post-surgery (mean = 2 months, SD = 1.73) and were grouped as the early post-surgery group, whereas, four participants (two females, two males) were between 41 and 43 months post-surgery (mean = 42 months, SD = 10.12) and were grouped as the late post-surgery group. Surgeries were performed at Brigham and Women's Hospital. Subjects in the early post-surgery group received osteomyocutaneous transplantations of the mid-face which included facial tissue and musculature, the nose, mandible, upper and lower lips. Bilateral buccal and marginal mandibular branches of the facial nerve coaptations were completed for this group. In the late post-surgery group, three of the subjects received myocutaneous full facial transplantations which included facial tissue and musculature including the upper and lower lips. One subject in the late post-surgery group received an osteomyocutaneous full transplantation which included facial tissue and musculature, the nose, maxilla, upper and lower lips. One of these subjects received bilateral frontal, zygomatic, buccal, and marginal mandibular branches of facial nerve coaptation. One received unilateral frontal, zygomatic, buccal, and marginal mandibular branches of facial nerve coaptation and unilateral temporal and cervical branch coaptation. Two received bilateral buccal and marginal mandibular branch of facial nerve coaptation. Patients in the early group were seen by the speech-language pathologist following surgery for the management of speech and swallowing deficits, including diet modifications and communication strategies (repetition, writing, text to speech) as necessary. For various reasons, including patient proximity to the hospital, social support systems and patient preference, at the time of these assessments, no patient had received consistent speech therapy targeting speech deficits. The study was approved by the Institutional Review Board of Partners HealthCare and all subjects provided written informed consent to participate in the study. Table 1 summarizes each participant's demographic and clinical information.

**Table 1:** Participants' demographic and clinical information.

	**Participant**	**Sex**	**Age**	**Type of facial transplantation**	**Months post-surgery**	Speech severity^*^
Early Post-surgery	P01	Male	60	Osteomyocutaneous	0	84
	P02	Male	33	Osteomyocutaneous	3	57
	P03	Male	38	Osteomyocutaneous	3	N/A
Late Post-surgery	P04	Female	47	Myocutaneous	43	14
	P05	Male	28	Myocutaneous	42	17
	P06	Male	33	Myocutaneous	41	11
	P07	Female	61	Osteomyocutaneous	48	N/A

* *Speech severity was rated on a scale of 0–100 with 0 representing normal and 100 representing profoundly severe. N/A was assigned to participants whose audio recordings had poor quality and, hence were not used in the perceptual assessment of speech severity*.

### Speech Samples

Participants were instructed to produce a total of 19 repetitions of the sentence “Buy Bobby a puppy” in four different speech modifications that varied in the degree of loudness (intensity) or rate. Because the utterance was produced on one breath, the likelihood of pauses occurring was low. Ten of the repetitions were produced at a normal rate and loudness, and three repetitions were produced at each of the three speech modifications loud, fast, and slow. The production of speech samples in the four speech modifications were blocked by task (each speech modification) and were presented in a fixed order. Audio recordings were collected using a lapel microphone (Model Countryman B3P4FF05B) with a sampling frequency of 44,100 Hz located approximately 15 cm from the participant's mouth. The duration of each task was measured to validate task performance (i.e., presumably, a slow task should take a longer time to complete and a fast task should take shorter time to complete).

### Speech Severity of the Participants

Audio recordings of five subjects were used to assess speech severity by two expert listeners (i.e., speech language pathologists) using the visual analog scale ranged from 0 (normal) to 100 (profoundly severe). Subjects showed varying levels of speech severity based on the perceptual ratings of the two listeners. The perceptual paradigm for clinical rating of speech severity will be discussed in Section Clinician Ratings of Speech Severity and Clarity. Two subjects in the early post-surgery group, P01, and P02, exhibited speech severity ratings of 84 and 57. Three subjects in the late post-surgery group, P04, P05, and P06 demonstrated speech severity of 14, 17, and 11, respectively. The quality of audio recordings obtained from participants P03 and P07 was poor, thereby, perceptual assessment of speech severity was not feasible for these participnats. The poor quality of the audio recordings was due to the ambient noise and assessor cross-talk, which can be avoided if audio samples are recorded in a laboratory setting, rather than clinical setting.

### Biomechanical Assessment

**Figure 1:**
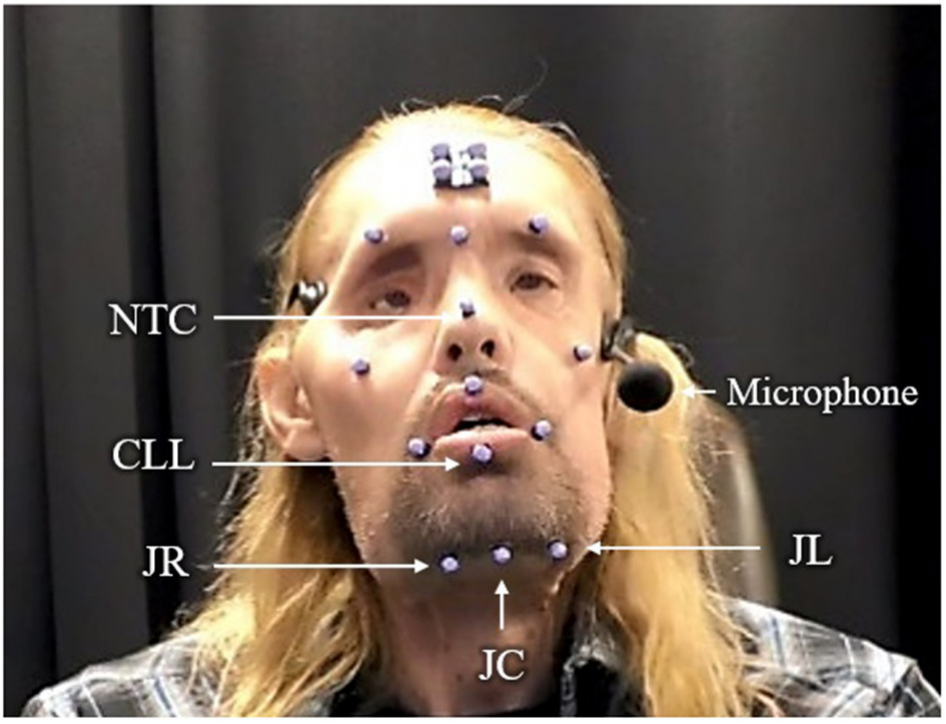
Placement of retroreflective markers on a participant's face.

Movement traces were recorded using an eight-camera 3D optical motion capture system (Motion Analysis, Rohnert Park, CA). An array of 17 retroreflective facial markers were positioned on different locations of participants' faces following a standard procedure (Figure 1). We limited our kinematic analyses to data obtained from the jaw and lips because these structures are primer movers during speech–unlike the cheeks, which are more active during facial expressions.

**Figure 2:**
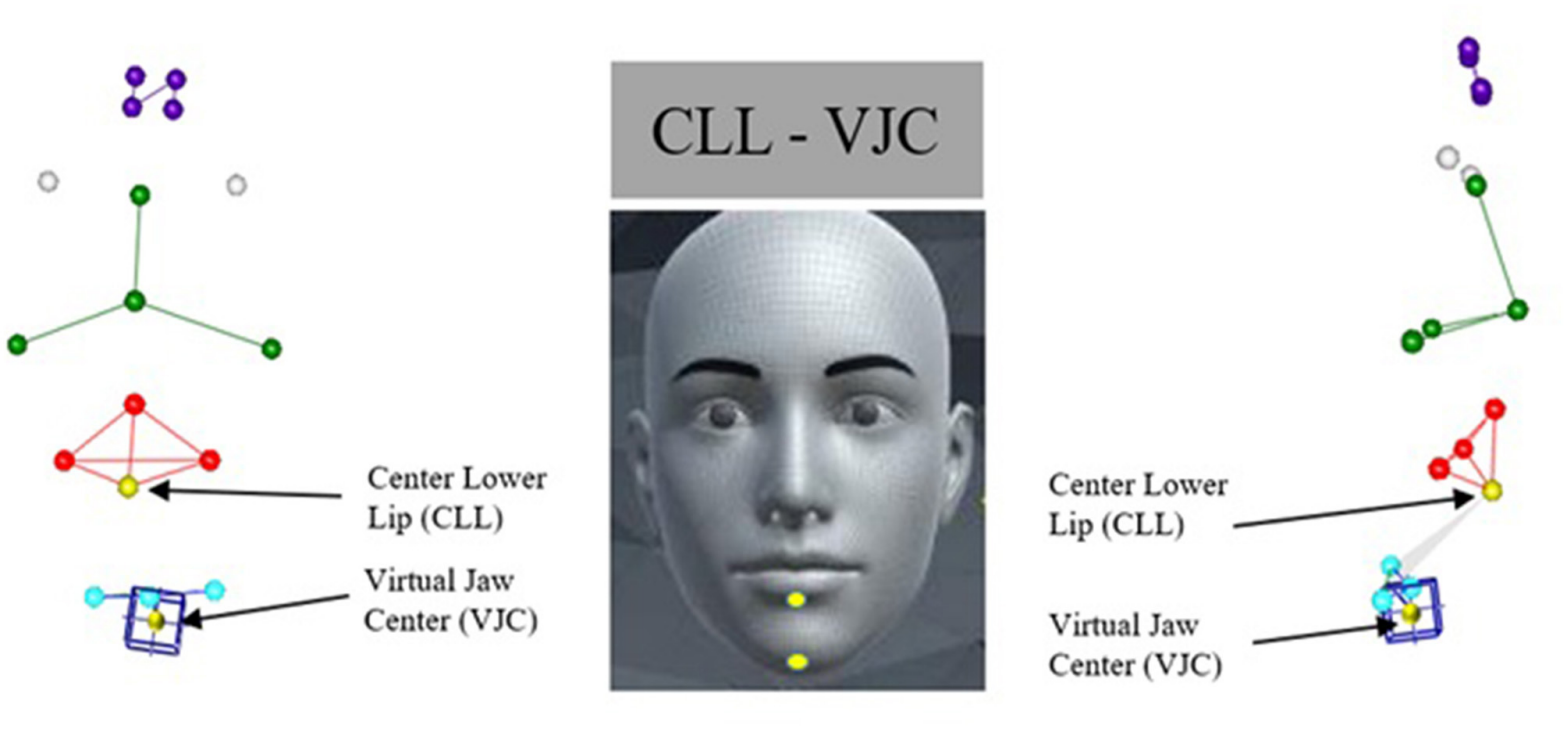
Left and right panels show 3D reconstruction of marker array in frontal and sagittal planes, respectively. Middle panel shows approximate anatomic location of the targeted markers used in this study, lower lip (CLL) and center chin (VJC). Virtual marker located midline chin is denoted with blue box.

Kinematic measures were extracted from three markers: the NTC marker which stands for “nose top center” and refers to the marker at the top of the nose dorsal bridge; the CLL marker which stands for “center lower lip” and refers to the midline lower lip marker; and the VJC marker which stands for “virtual jaw center” and refers to the virtual midline jaw marker. The NTC, CLL, and VJC acronyms were created by our lab to label the corresponding markers on the face. To distinguish the contribution of jaw-driven lower lip movement from lower lip autonomous movement during production of speech tasks, movement of CLL was represented in two ways: one that included movements of the underlying jaw (lower lip + jaw) and one that was independent from the movements of the jaw (lower lip - jaw). In the first approach, the Euclidean distance between NTC to CLL markers was measured to calculate kinematic properties of the combined movement of the lip and jaw lip movement (i.e., lower lip + jaw). In the second approach (i.e., lower lip - jaw), a virtual marker for the center of the jaw was calculated in CORTEX (Motion Analysis, Rohnert Park, CA) as the linear distance between the right and left jaw markers protruded 30% perpendicular to the line that connects left and right lower lip markers. The VJC was consistently used for all participants because some patients with facial transplantation had facial hair on the chin which did not allow for the placement of a marker on the underside of the body of the mandible. Subsequently, the Euclidean distance between the VJC and the center of lower lip was measured to calculate the kinematic measures of the lip movement independent of the jaw (Figure 2).

A four-sensor head marker was used to subtract head movement (translation and rotation) from the facial markers. Motion capture recordings were cut and labeled using the CORTEX Motion Analysis software (Motion Analysis, Rohnert Park, CA). To extract kinematic measures, the initial segmentation landmark was placed on the first trough associated with the lip closure for /b/ in /bai/ and the final landmark was placed on the last trough associated with the second /p/ in /p*a*pi/. The outcome kinematic measures, thus, included all bilabial closures and vowels averaged together between the two segmentation landmarks. Subsequently, the data were transferred to SMASH, a customized MATLAB-based software program (50), to calculate two kinematic measures of average speed of movement (mm/s) and range of movement (mm^3^) from the movement time series of the lower lip independent of the jaw. Each 3D positional time series was represented as the 3D Euclidean distance between the markers. Average speed was calculated as the average value in the first derivative of the 3D Euclidean distance movement time history. Range of motion was measured by the change in distance in mm between the maximum opening and maximum closing positions during speech. Values obtained across the repetitions of the “Buy Bobby a puppy” in each speech modifications were averaged.

### Clinician Ratings of Speech Severity and Clarity

Two speech-language pathologists rated the speech severity and speech clarity of five participants (two from the early post-surgery group and three from the late post-surgery group) who had good quality audio recordings. The speech stimuli were the same samples (i.e., “Buy Bobby a puppy”) from which the kinematic measures were extracted. Perceptual evaluation of speech severity and speech clarity were conducted separately. Two different listening paradigms were designed for the perceptual judgment of speech severity and speech clarity using a computerized continuous visual analog scale (VAS). Written instructions were provided for each paradigm and each speaker completed the perceptual tasks blindly and independently.

### Ratings of Speech Severity

Clinical ratings were performed to index the overall severity of speech impairment in individuals at early and late stages of neural recovery. These measures are important for documenting the range of impairment in our cohort and provide a metric for evaluating the potential effects of baseline severity and response to speech modifications. For speech severity, listeners were instructed to rate the overall speech naturalness and prosody, resonance and voice qualities, and articulatory precision. The speech severity paradigm consisted of five blocks (one block for each subject) presented to each listener in a random order. Each block included three repetitions of the sentence “Buy Bobby a puppy” in normal speech produced by a study participant, and the same sentence produced by a normal speaker of the participant's same sex as the reference sample. Speech stimuli in each block were also presented to the listeners in a random order. Listeners were asked to listen to the reference sample prior to the rating of each stimulus and using the computer mouse, drag the corresponding slider vertically anywhere along a continuous 100 mm scale (0 for normal severity and 100 profound severe) to indicate their responses. Each listener was able to listen to each stimulus up to five times. Upon the completion of the listening tasks for speech severity, the program converted responses to numerical values ranging from 0 (normal) to 100 (profoundly severe).

The average intraclass correlation coefficient (ICC) between the ratings of the listeners was 0.99 (*p* = 0.0001) and the single measures ICC was 0.98 (*p* = 0.0001), indicating excellent interrater reliability. The Pearson product correlation coefficients of ratings of speech severity ranged from 0.96 to 0.99 for the Listener 1, with a mean of 0.97 (SD = 0.015). For Listener 2, correlations ranged from 0.96 to 0.99 with a mean of 0.98 (SD = 0.015).

### Ratings of Speech Clarity

Clinical ratings of speech clarity were conducted to compare the effectiveness of different behavioral speech modifications in deriving a clearer-than-normal speech (aim 2 of the study). Speech clarity refers to how well and clear speech samples are enunciated and can be assessed in both connected and isolated speech utterances. Speech intelligibility, on the other hand, is defined as how well-speech samples are understood and is usually assessed in connected speech. Because our participants did not have severe speech impairments and had high baseline intelligibility, speech clarity is the preferred metric of functional speech as ceiling effects would render intelligibility data unusable.

For speech clarity, listeners were instructed to rate how clear and well-enunciated the speech sample in a given speech modification is relative to the same sample produced in a normal speech. The listening paradigm for speech clarity also consisted of five blocks (one for each subject) that were randomly presented to the listeners. Each block included 10 speech stimuli: three repetitions of the sentence “Buy Bobby a puppy” in each of the fast, loud, and slow speech modifications and the same sentence in normal speech as the reference sample. The scale for VAS was a 100 mm continuum ranged from −50 to 50. The speech clarity of the reference sample was set as 0 (baseline). Listeners were instructed to replay the reference sample prior to the rating of each stimulus and drag the corresponding slider vertically anywhere above the baseline 0 if they judged that the speech clarity of the stimulus in a given speech modification is improved relative to the reference or drag the slider below the baseline 0 if the clarity decreased relative to the reference stimulus. Similar to the speech severity paradigm, listeners were able to listen to each stimulus up to 5 times. If listeners needed to replay a sample to make perceptual judgement, they were instructed to replay the reference sample prior to the rating of the stimulus in each repetition. Therefore, in each repetition, they basically compare the clarity of the sample to the reference sample rather than replaying the sample multiple times. The combined presentation of the reference (auditory anchor) and the speech sample has been implemented in the previous perceptual studies and have been shown to significantly increase the effectiveness and reliability of the perceptual judgment (51–53). Although we did not keep track of the number of listening attempts, listeners rarely listened to the samples five times. Upon the completion of the listening tasks for speech clarity, the program converted responses to numerical values ranging from −50 to 50.

### Statistical Analysis

Separate one-way analysis of variance (ANOVA) with Tukey *post hoc* tests were used for early and late post-surgery groups to compare the duration of speech samples in the four speech modifications. Mann-Whitney *U* tests were used to compare the average speed and range of lower lip movement in the early and late post-surgery groups across the four speech modifications (between-group comparisons). Additionally, to compare the lip articulatory performance across speech modifications, Mann-Whitney U tests were performed separately for each pair of speech modifications in each group (within-group comparisons). Finally, descriptive statistics for listeners' ratings of the speech severity and clarity of the “Buy Bobby a puppy” sentence produced by five subjects were calculated to perceptually identify (1) the speech modification with potential to derive articulatory precision and clear speech and (2) the degree to which participants were able to adapt their motor system to various articulatory demands of speech modifications. The intraclass correlation coefficient (ICC) test was applied to assess the consistency between speech clarity ratings of the two listeners, using a two-way mixed effects. Intrarater reliability was examined using Pearson correlation product. All statistical analyses were performed in SPSS statistical software version 25 and the α-level of 0.05 was set as the level of significance. Bonferroni corrections were applied to manage family-wise multiple comparisons.

## Results

### Task Effects on Speech Duration

**Figure 3:**
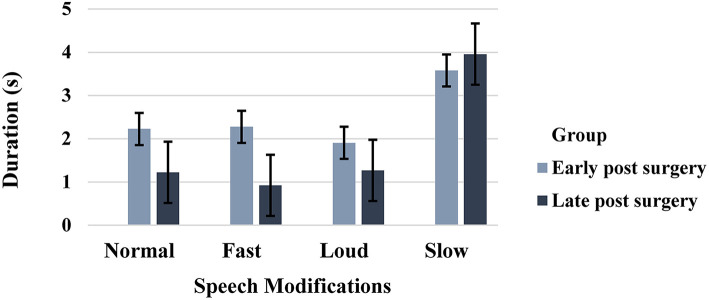
Duration (s) of speech samples produced in normal, fast, loud, and slow speech modifications in early and late post-surgery groups (error bars represent the standard error (SE) of the mean).

Among the four speech modifications, speech samples produced in the slow speech modification were the longest in duration compared to the other speech modifications in both early and late post-surgery groups. In addition, speech productions in the fast speech modification were the shortest in duration as expected in individuals in the late post-surgery group. Individuals in the early post-surgery group, however, were observed to produce speech productions in the loud speech modification in the shortest duration of time (Figure 3). Results of one-way ANOVA indicated significant differences across the duration of speech samples produced by individuals in the early post-surgery group (*p* = 0.03) as well as individuals in the late post-surgery group (*p* = 0.0001). Tukey *post hoc* tests revealed that individuals in the late post-surgery group produced samples in the slow speech modification significantly longer than samples in normal, fast, and loud speech modifications (*p* = 0.0001). Individuals in the early post-surgery group, exhibited significant differences between the duration of samples produced in slow and normal speech modifications (*p* = 0.03) and between the duration of samples produced in slow and loud speech modifications (*p* = 0.04).

### Between-Group Comparisons (Early vs. Late Post-surgery)

**Figure 4:**
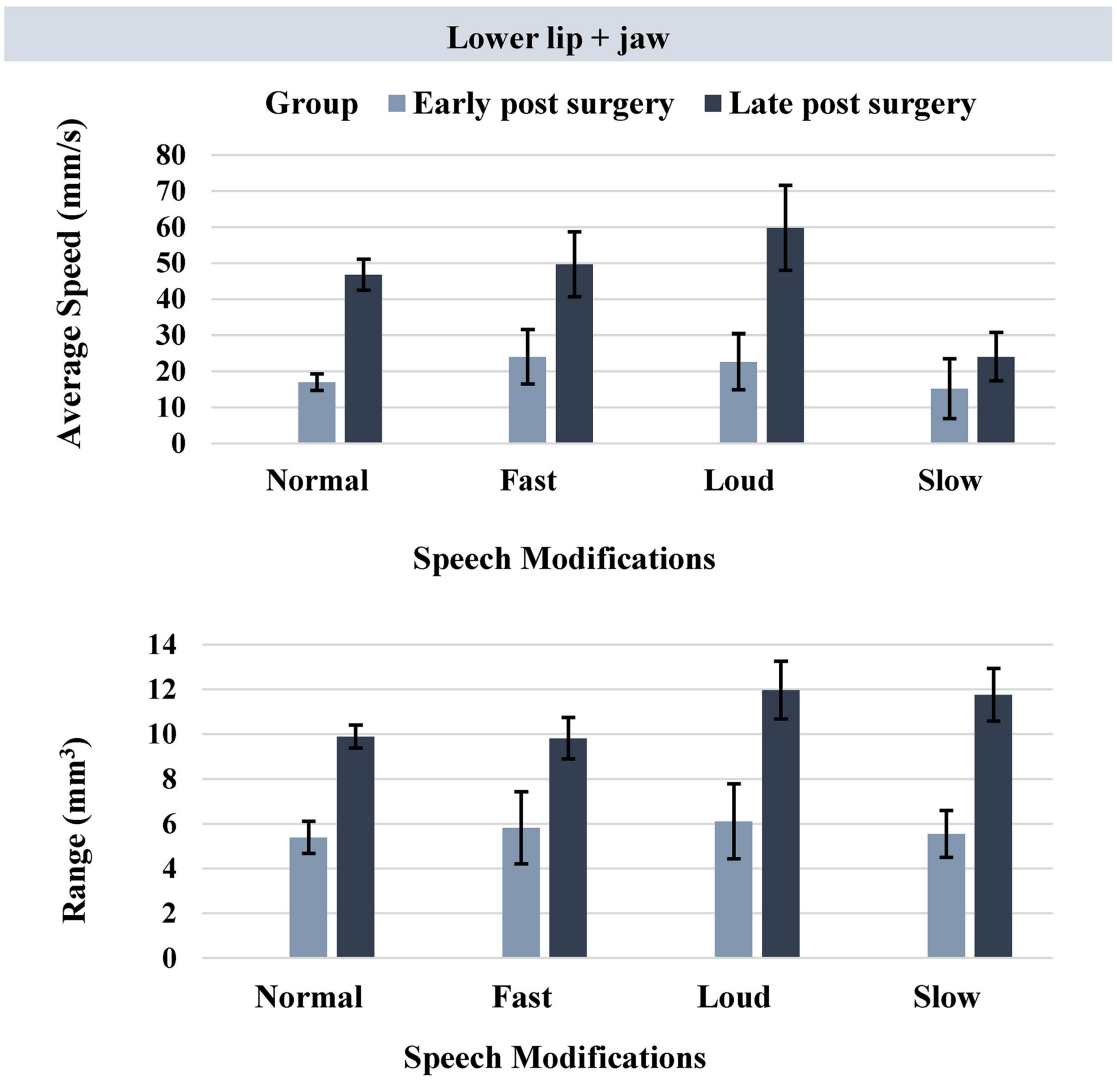
Bar plots representing the average speed (mm/s) and range (mm^3^) of lower lip + jaw movement in early and late post-surgery groups (error bars represent the standard error (SE) of the mean).

For kinematic measures extracted from the lower lip + jaw movement, the late post-surgery group had a significantly (adjusted *p*-value = 0.0125) faster speed and larger range of movement compared to those in the early post-surgery group across all speech modifications except slow (Table 2, Figure 4).

**Table 2:** Comparison of average speed (mm/s) and range (mm^3^) of lower lip+ jaw movement in early and late post-surgery groups.

**Kinematic measure**	**Speech modification**			**Mann-Whitney *U* test** ***P*-value**
**lower lip + jaw**		**Early post surgeryMean (SD)**	**Late post surgery Mean (SD)**	
Average speed(mm/s)	Normal	17.01 (6.26)	46.75 (13.44)	0.0001
	Fast	24.01 (9.81)	49.66 (14.14)	0.0001
	Loud	22.64 (9.30)	59.79 (15.34)	0.0001
	Slow	15.21 (10.76)	24.06 (11.05)	0.110
Range of movement(mm^3^)	Normal	5.39 (1.92)	9.89 (1.63)	0.0001
	Fast	5.82 (2.09)	9.82 (1.46)	0.0001
	Loud	6.11 (2.01)	11.97 (1.68)	0.0001
	Slow	5.55 (1.37)	11.76 (1.94)	0.0001

*Gray areas represent significant comparisons at the adjusted p-value (α = 0.0125)*.

**Figure 5:**
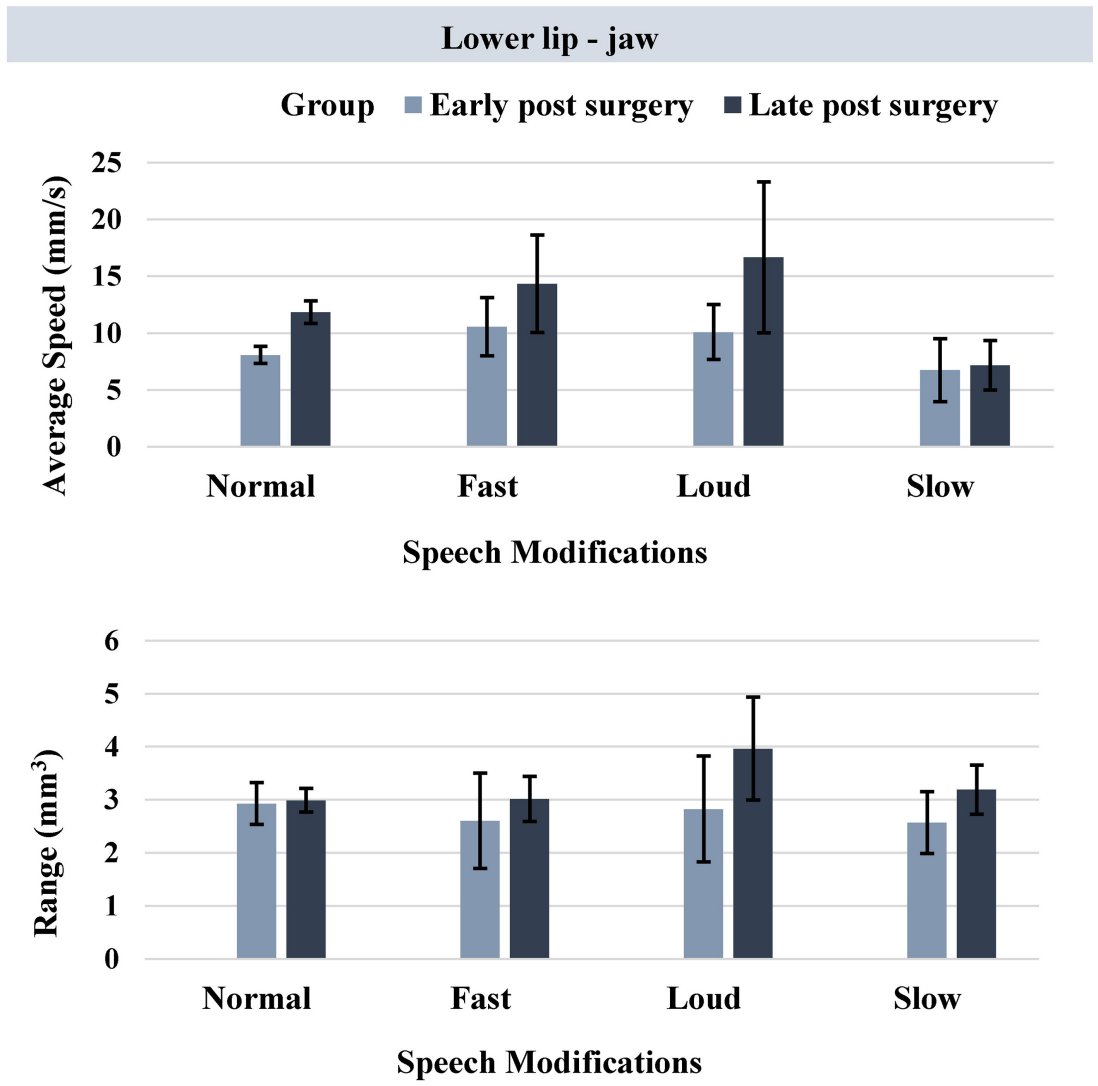
Bar plots representing the average speed (mm/s) and range (mm^3^) of lower lip - jaw movement in early and late post-surgery groups (error bars represent the standard error (SE) of the mean).

For kinematic measures extracted from the lower lip – jaw movement, Mann-Whitney *U* tests revealed a significantly greater average speed of movement in the late post-surgery group compared to the early post-surgery group (adjusted *p*-value = 0.0125). The observed between-group difference was during the normal speech only (Table 3, Figure 5).

**Table 3:** Comparison of average speed (mm/s) and range (mm^3^) of lower lip - jaw movement in early and late post-surgery groups.

**Kinematic measure**	**Speech modification**			**Mann-Whitney *U* test** ***P*-value**
**lower lip - jaw**		**Early post surgeryMean (SD)**	**Late post surgery Mean (SD)**	
Average speed (mm/s)				
	Normal	8.10 (2.02)	11.87 (3.07)	0.0001
	Fast	10.59 (3.34)	14.35 (5.55)	0.136
	Loud	10.13 (2.89)	16.69 (6.32)	0.059
	Slow	6.77 (3.61)	7.19 (3.06)	0.243
Range of movement(mm^3^)	Normal	2.93 (1.06)	2.99 (0.69)	0.260
	Fast	2.60 (1.17)	3.02 (0.55)	0.190
	Loud	2.83 (1.19)	3.97 (0.92)	0.081
	Slow	2.57 (0.76)	3.19 (0.64)	0.079

*The gray area represents the significant comparison at the adjusted p-value (α = 0.0125)*.

### Within-Group Comparisons (Task Effect)

In the early post-surgery group, no significant differences were observed across the speech modifications using kinematic measures extracted from the lower lip ± jaw movement. In the late post-surgery group, however, significant between-task differences were observed using kinematic measures extracted from both the lower lip + jaw movement and lower lip - jaw movement (Table 4).

**Table 4:** Pairwise comparisons of speech modifications in terms of kinematic measures extracted from lower lip ± jaw movement in the late post-surgery group.

**Kinematic measure**	**Pairwise comparison**	**Mann-Whitney** ***U*** **test** ***P*****-value**	
		**lower lip + jaw movement**	**lower lip - jaw movement**
Average speed(mm/s)	Normal vs. Fast	0.550	0.339
	Normal vs. Loud	0.007	0.075
	Normal vs. Slow	<0.001	0.000
	Fast vs. Loud	0.219	0.328
	Fast vs. Slow	<0.001	0.001
	Loud vs. Slow	<0.001	0.002
Range of movement(mm^3^)	Normal vs. Fast	0.931	0.810
	Normal vs. Loud	0.003	0.017
	Normal vs. Slow	0.004	0.436
	Fast vs. Loud	0.009	0.036
	Fast vs. Slow	0.011	0.604
	Loud vs. Slow	0.948	0.093

*Gray areas represent significant comparisons at the adjusted p-value (α = 0.008)*.

### Speech Clarity

**Figure 6:**
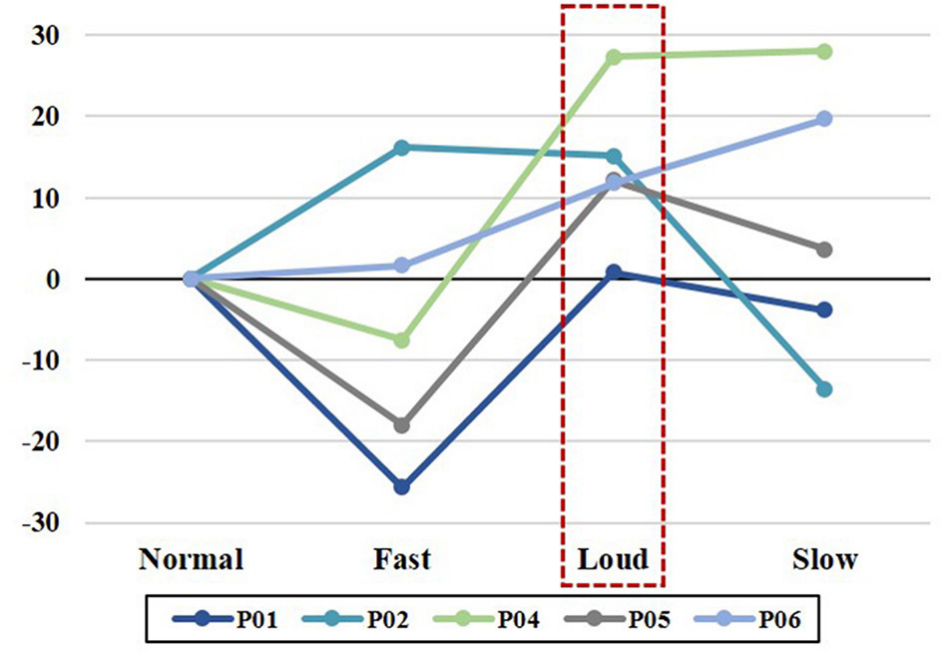
Perceptual ratings of speech clarity in fast, loud, and slow conditions relative to the normal speech in five participants. P01 and P02 participants belong to the early post-surgery group and the three other participants (P04, P05, and P06) belong to the late post-surgery group.

Qualitative analyses of listener's judgment of speech clarity indicated that among fast, loud, and slow speech modifications, speech loudness consistently improved speech clarity compared to the baseline (speech clarity of normal speech) in the five subjects incorporated in the perceptual component of the study (Figure 6). In this figure, P01 and P02 belong to the early post-surgery group and the three other participants (P04, P05, and P06) belong to the late post-surgery group. The baseline 0 was assigned to the clarity of speech stimuli produced in the normal speech (i.e., reference). Accordingly, scale values above baseline 0 indicate relatively better speech clarity, whereas values below the baseline 0 represent relatively poorer speech clarity. Slowed speech improved speech clarity in participants in the late post-surgery group but decreased speech clarity in participants in the early post-surgery group. Rating of speech clarity across the three speech modifications (fast, loud, and slow) indicated that participants in the early post-surgery group (P01 and P02) showed improvement in speech clarity about eight units on the scale for the loud speech modification. In this group, the speech clarity decreased about five and nine units on the scale for the fast and slow speech modifications, respectively. In the late post-surgery group (P04, P05, and P06), the average rating of speech clarity across the three speech modifications (fast, loud, and slow) indicated improved speech clarity (17 units on the scale) for the loud and slow speaking modes and decreased speech clarity (eight units on the scale) for the fast speaking mode.

For the observed perceptual ratings of speech clarity, the average intraclass correlation coefficient (ICC) was 0.75 (*p* < 0.05) and the single measures ICC was 0.74 (*p* < 0.05), indicating fair interrater reliability. The average Pearson product correlation coefficients of ratings of speech clarity across fast, loud, and slow speech modifications for listener 1 and 2 were 0.88 (*p* < 0.05) and 0.70 (*p* < 0.05) respectively. Table 5 summarizes the average of speech clarity ratings performed by each listener for fast, loud, and slow speech modifications in reference to the normal speech (baseline 0).

**Table 5:** The average of speech clarity ratings by listeners 1 and 2 for participants in early (P01 and P02) and late (P04, P05, and P06) post-surgery groups during fast, loud, and slow speech modifications.

	**Early post-surgery Group**			**Late post-surgery Group**		
	**Fast**	**Loud**	**Slow**	**Fast**	**Loud**	**Slow**
Listener 1	−5	3	−10	−8	13	5
Listener 2	−5	13	−8	−8	21	30

*Ratings are in reference to the normal speech (baseline 0)*.

## Discussion

The purpose of this study was to determine (1) the extent to which patients recovering from facial transplantation surgery can adapt their motor system to various articulatory demands of different speech modification using measures of facial biomechanics, and (2) the comparative effects of these adaptations on speech clarity. Results from our study suggest that (1) motor adaptation to articulatory demands of speech modifications increased from 2 to 42 months post-surgery as an indication of neural recovery; (2) across the four speech modifications, loud speech modification most consistently improved speech clarity during early and late stages of neuromotor recovery; and (3) individuals in early and late stages of neuromotor recovery over-rely on jaw for the lip articulatory function across all speech modifications. These findings help improve our understanding of underlying mechanisms of motor speech recovery following facial transplantation and offer a speech modification strategy that may help improve speech intelligibility in this patient population particularly in the early stages of recovery.

### Facial Motor Capacity for Speech Demands Increased From 2 to 42 Months Post-surgery

Our participants' ability to accommodate the varying motoric demands required by the speech modifications significantly improved from early (~2 months) to late (~42 months) post-surgery. Restricted facial motor capacity in the early post-surgery group was demonstrated by (1) the lack of difference in the duration of speech samples produced by this group in normal, fast, and loud speech modifications, and (2) no differences between speed and range of lower lip movement during these various speech modifications. In contrast, individuals in the late post-surgery group were able to make modification-specific articulatory adjustments in speed and range of lip movement, as the magnitude of speed and range of lip movement during production of all speech modifications were significantly larger in the late post-surgery group compared to values of their corresponding modification in the early post-surgery group. Improvement in facial motor function in the later stage of neural recovery supports findings reported by Grigos et al. (7), who observed significant increase in jaw displacement and lip aperture in the vertical plane over a 13-month period for nonspeech and speech tasks produced by a single facial transplant patient. These findings are also consistent with the prior longitudinal case study conducted by De Letter et al. (2) in which improved functional neuromotor recovery were observed up to 38 months post-surgery in a patient who underwent facial allotransplantation.

### Loud Speech Modification Consistently Improved Speech Clarity During Early and Late Stages of Neuromotor Recovery

Perceptual evaluations of speech produced during normal, fast, loud, and slow speech modifications in our study demonstrated that relative to normal speech, increased speech loudness resulted in enhanced speech clarity across the recovery spectrum. Additionally, for those is the late-recovery group, slow speech resulted in improved speech clarity. These findings are supported by prominent gains in range of lip movements observed during the loud and slow speech modifications in our study. Consistent gain in speech clarity during loud speech support previous findings as well as the LSVT^R^ program that advocate loud speech intervention to improve speech motor system and overall intelligibility in populations with dysarthria (8, 9, 12, 37, 54). These findings provided empirical evidence for potential benefits of implementing loud speech modification as an intervention technique to enhance clarity and most possibly intelligibility in individuals with facial transplantation. More studies with interventional research design are warranted to test the efficacy of loud speech on speech intelligibility in individuals post facial transplant surgery.

### Individuals in Early and Late Stages of Neuromotor Recovery Over-Rely on Jaw for the Lip Articulatory Function Across All Speech Modifications

Although all subjects in the early-post surgery group and one subject in the late post-surgery group underwent osteomyocutaneous transplantations, jaw-dependent lip movements outperformed jaw-independent lip movements in deriving articulatory distinctions between groups and across speech modifications, as kinematic measures extracted from lip + jaw movement showed greater differences between speech modifications than the ones obtained from the lip – jaw movement. In addition, greater between-group differences were captured using kinematic measures extracted from lip + jaw movement, rather than the lip – jaw movement.

Unlike the lip + jaw movement, which demonstrated significant improvement in speed and range of movement across all speech modifications over the course of neuromotor recovery, lip movements that were decoupled from those of the jaw (lip - jaw) were similar regardless of the type of speech modification in both early and late post-surgery groups. For the purposes of functional speech, it appears that neuromotor recovery of the lips is limited, at least for the participants who participated in this study. In the absence of recovery of lip motor control, individuals with face transplant may depend on the jaw as an articulatory strategy to enhance speech function. These findings provide value when considering functional outcomes for potential surgical candidates, as patients requiring surgical techniques that impede jaw movement may experience worse functional speech outcomes than those who do not. Similar findings have been reported in populations with impaired speech motor control such as multiple sclerosis (55) and amyotrophic lateral sclerosis (56, 57) or during normal speech motor development in neurotypical children (58).

## Conclusion

Our findings suggest that the articulatory kinematic adaptations to speech modifications are significantly restricted in individuals at the early stage of neuromotor recovery post facial transplant surgery but improve over time. In addition, our findings provide empirical evidence for the overreliance of the jaw to support lip articulatory functions during speech over the course of neural recovery. Despite speech motor restrictions on speech modifications and jaw reliance during speech, loud speech may result in increased lip and jaw movement and increased speech clarity as early as 2-months post-surgery, and therefore, may be beneficial as a behavioral speech modification to improve functional speech in this population.

Two limitations of this study need to be acknowledged. First, the sample size in each group was small. Second, the elicitation procedure for speech modification tasks was not in a random order. Future studies with larger sample size and longitudinal design are warranted to further substantiate the findings of this study. It should be noted that the current study examined the effect of speech modifications on articulatory movements over one session, and results cannot be over-interpreted as treatment outcomes of loud or slow speech modifications. Further work is required to understand the effect of these approaches when applied during intervention and to identify speech kinematic profiles of speakers who benefit from different speech treatment approaches. In addition, in this study, speech samples were not produced in clear speech modification (i.e., hyperarticulated speech). Given that several perceptual studies have shown significant improvement to speech intelligibility during clear speech compared to normal speech (10, 59–62), future studies are encouraged to implement cues for clear speech and compare the effectiveness of that to those of loud and slow speech modifications.

## Data Availability Statement

The raw data supporting the conclusions of this article will be made available by the authors, without undue reservation.

## Ethics Statement

The studies involving human participants were reviewed and approved by Partners health care IRB. The patients/participants provided their written informed consent to participate in this study. Written informed consent was obtained from the individual(s) for the publication of any potentially identifiable images or data included in this article.

## Author Contributions

ME had a major role in study conceptualization and design, data analyses, interpretation of the findings, and writing the manuscript. BJP contributed to data collection and interpretation of the findings, reviewed the manuscript, and provided feedback. BR contributed to data collection and data analyses. HV contributed to data analyses. BP reviewed the manuscript and provided feedback. JG had a major role in the study conceptualization and design, interpretation of the findings, and manuscript preparation. All authors contributed to the article and approved the submitted version.

## Conflict of Interest

The authors declare that the research was conducted in the absence of any commercial or financial relationships that could be construed as a potential conflict of interest.
